# Effect of Ghrelin on Mortality and Cardiovascular Outcomes in Experimental Rat and Mice Models of Heart Failure: A Systematic Review and Meta-Analysis

**DOI:** 10.1371/journal.pone.0126697

**Published:** 2015-05-27

**Authors:** Mahalaqua Nazli Khatib, Anuraj Shankar, Richard Kirubakaran, Kingsley Agho, Padam Simkhada, Shilpa Gaidhane, Deepak Saxena, Unnikrishnan B, Dilip Gode, Abhay Gaidhane, Syed Quazi Zahiruddin

**Affiliations:** 1 Department of Physiology, Datta Meghe Institute of Medical Sciences, Wardha, Maharashtra State, India; 2 Department of Nutrition, Harvard School of Public Health, Harvard University, Cambridge, Massachusetts, United States of America; 3 South Asian Cochrane Centre, Christian Medical College, Vellore, India; 4 Department Biostatistics, University of Western Sydney, Sydney, Australia; 5 Centre for Public Health, Liverpool John Moores University, Liverpool, United Kingdom; 6 Department of Medicine, Datta Meghe Institute of Medical Sciences, Wardha, Maharashtra State, India; 7 Indian Institute of Public Health-Gandhinagar, Public Health Foundation of India, New Delhi, India; 8 Department of Community Medicine, Manipal University, Manipal, India; 9 Datta Meghe Institute of Medical Sciences, Wardha, Maharashtra State, India; 10 Department of Community Medicine, Datta Meghe Institute of Medical Sciences, Wardha, Maharashtra State, India; Temple University, UNITED STATES

## Abstract

**Background:**

Heart failure (HF) continues to be a challenging condition in terms of prevention and management of the disease. Studies have demonstrated various cardio-protective effects of Ghrelin. The aim of the study is to determine the effect of Ghrelin on mortality and cardiac function in experimental rats/mice models of HF.

**Methods:**

Data sources: PUBMED, Scopus. We searched the Digital Dissertations and conference proceedings on Web of Science. Search methods: We systematically searched for all controlled trials (upto November 2014) which assessed the effects of Ghrelin (irrespective of dose, form, frequency, duration and route of administration) on mortality and cardiac function in rats/ mice models of HF. Ghrelin administration irrespective of dose, form, frequency, duration and route of administration. Data collection and analysis: Two authors independently assessed each abstract for eligibility and extracted data on characteristics of the experimental model used, intervention and outcome measures. We assessed the methodological quality by SYRCLE’s risk of bias tool for all studies and the quality of evidence by GRADEpro. We performed meta-analysis using RevMan 5.3.

**Results:**

A total of 325 animals (rats and mice) were analyzed across seven studies. The meta-analysis revealed that the mortality in Ghrelin group was 31.1% and in control group was 40% (RR 0.83, 95% CI 0.46 to 1.47) i.e Ghrelin group had 68 fewer deaths per 1000 (from 216 fewer to 188 more) as compared to the control group. The meta-analysis reveals that the heart rate in rats/mice on Ghrelin was higher (MD 13.11, 95% CI 1.14 to 25.08, P=0.66) while the mean arterial blood pressure (MD -1.38, 95% CI -5.16 to 2.41, P=0.48) and left ventricular end diastolic pressure (MD -2.45, 95% CI -4.46 to -0.43, P=0.02) were lower as compared to the those on placebo. There were insignificant changes in cardiac output (SMD 0.28, 95% CI -0.24 to 0.80, P=0.29) and left ventricular end systolic pressure (MD 1.48, 95% CI -3.86 to 6.82, P=0.59).

**Conclusions:**

The existing data provides evidence to suggest that Ghrelin may lower the risk of mortality and improve cardiovascular outcomes. However; the quality of evidence as assessed by GRADEpro is low to very low. Clinical judgments to administer Ghrelin to patients with HF must be made on better designed animal studies.

## Background

Heart failure (HF) is associated with considerable morbidity and mortality [1]. More than one million patients are hospitalized annually with a primary diagnosis of HF which accounts to extensive use of health care resources [2, 3]. It is one of the most common cause for hospital admissions for elderly patients and still continues to be one of the most challenging condition in terms of prevention and management of the disease [4]. Due to optimized treatment of acute myocardial infarction; there is an increase in the number of patients surviving with severely deteriorated cardiac function. Despite improved treatment; HF still has a very bad prognosis and is accompanied by decreased quality of life and considerable health care cost [4, 5].

A 28-amino acid polypeptide; Ghrelin was first identified in 1999 by Kojima et al in gastric cells of rats [6]. Because of widespread distribution of its downstream receptor; Growth hormone secretagogue receptor (GHSR-1a); Ghrelin fulfils an important role in a number of physiological actions including hormonal, cardio-respiratory, metabolic, immunological, and other actions [7–14]. The versatile nature of Ghrelin makes it an interesting intervention for the treatment of various diseases and conditions [12]. Even though Ghrelin was initially believed to be solely involved in regulation of energy balance and maintenance of body weight; it is recently hypothesized that the cardiovascular system is also its important target [9, 15–17]. GHSR-1a is extensively distributed in coronary arteries, aorta, myocardium, and veins [9]; indicating that this gut hormone can also exert direct action upon cardiovascular tissues. Several experimental studies have investigated the potential role for Ghrelin in the treatment of heart failure [18–21]. Ghrelin has been shown to lower peripheral resistance, either directly at the vascular level or by modulating sympathetic nervous activity [9]. Additional actions include enhancement of contractility and anti-inflammatory effects [22]. Injecting Ghrelin has also been demonstrated to enhance exercise capacity, improve left ventricular function, improve endothelial function, increase myocardial contractility, inhibit myocardial cell apoptosis, and preserve cardiac function after myocardial infarction [19–21, 23–32]. Other cardio-protective effects of Ghrelin include reduction of arterial blood pressure, protection from ischemia/reperfusion injury, limitation of progression of atherosclerosis and improvement of prognosis of HF [8, 9, 33–37].

Positive and protective effects have been described in individual preclinical studies that could be of great benefit in the treatment of HF. We performed this review to determine the effect of administration of Ghrelin in preclinical rat/mice studies to inform evidence-based decision making for formulating new hypotheses for future (pre)clinical testing. Furthermore, there is a need to evaluate the optimal dosage, route and schedule of Ghrelin so as to plan a best possible therapy. The purpose of this review is to comprehensively appraise and analyze the current evidences regarding the efficacy of Ghrelin in treating HF.

Objective: To determine the effect of administration of Ghrelin on mortality and cardiac functions in rats and mice with experimentally induced heart failure.

## Methods

The Systematic Approach was conducted by following the methods of Cochrane systematic review [38] and reported in accordance with the PRISMA Guidelines (S1 Table) [39]. Institutional Ethics Committee of Datta Meghe Institute of Medical Sciences gave the approval of this study.

### Eligibility criteria

We considered following criterias for including studies in this review:

#### Type of studies

Controlled animal (rat or mice) studies (full text). We excluded studies published in non-peer reviewed journals.

#### Animal model

We included laboratory studies on rat/mice models of heart failure induced by either ligation of the coronary artery or by administration of Isoproterenol (ISO) regardless of the age or weight of the rats/mice.

#### Experimental interventions

Administration of Ghrelin at any dose or in any form was considered for inclusion irrespective of the frequency and duration of treatment.

#### Control group

We compared the effect of administration of Ghrelin with control/placebo.

### Types of outcome measures

The primary outcome of the study was to determine the effect of Ghrelin on mortality and secondary outcomes were parameters reflecting hemodynamic and cardiac function e.g. heart rate, mean arterial pressure, cardiac output, stroke volume, ejection fraction, Left Ventricular End Diastolic Pressure (LVEDP) and Left Ventricular End Systolic Pressure (LVESP).

### Search methods for identification of studies

We searched for all publications describing controlled trials of Ghrelin in rats/mice models of heart failure through electronic searches from 1999 (since Ghrelin was first isolated and identified by Kojima and Kangawa et al. in 1999) up to November 2014 on the PubMed, Scopus, CINAHL. We searched the Digital Dissertations and conference proceedings on Web of Science. The bibliographies of all include papers were screened for additional studies. We did not restrict the search to English language. We screened the reference lists of (systematic) reviews to locate additional primary studies that were not picked up by our search. To avoid missing studies in the search strategy, we gave consideration to spelling of terms used in different countries. The search strategy with PubMed was as in S2 Table). We scanned book chapters and editorials. We also conducted hand-searching for journals and conference proceedings.

### Screening and selection of studies

Two authors (SG and AG) independently assessed all potentially relevant trials according to the pre-specified selection criteria by screening the title and abstract. The studies were classified as relevant, irrelevant and unclear. All the irrelevant studies were discarded. We retrieved the full texts of all the potentially relevant and unclear studies to determine their eligibility with the help of a standardized inclusion form and any disagreement between the reviewers regarding the eligibility of a study was resolved by discussion with the third reviewer (AG).

### Data extraction and management

Two authors (MNK and SZQ) independently extracted the data using a pre-tested data extraction form and any disagreement between the authors was resolved through discussions. Following data was extracted:

1. Methodological quality of the study. Reporting of study design, method of randomization, total study duration, sequence generation, concealment of allocation, blinding of participants and other concerns about bias (investigators, attrition and reporting bias).
2. Characteristics of the animal model. Animal species, weight, age, gender, laboratory setting, sample size.
3. Intervention. For each intervention and control group we extracted the data separately as intervention, number of animals randomized to each treatment arm, dose, time of therapy, route of administration and duration of treatment.
4. Outcomes measures. Mortality, heart rate (HR), mean arterial blood pressure (MABP), cardiac output (CO), ejection fraction (EF), left ventricular end systolic pressure (LVESP) and left ventricular end diastolic pressure (LVEDP).

Disagreements amongst the reviewers were resolved by discussion. Wherever required, we consulted a third reviewer (SZQ) to resolve the disagreements.

### Assessment of risk of bias in included studies

We assessed the risk of bias (RoB) for each study with the help of SYRCLE’s RoB tool for animal studies [40]. Two authors independently (SG and PS) assessed the risk of bias of the included studies across domains of sequence generation (Selection bias), baseline characteristics (Selection bias), allocation concealment (Selection bias), random housing (Performance bias), blinding of personnel and outcome assessors (Performance and detection bias), random outcome assessment (Detection bias), incomplete outcome data (Attrition bias), selective outcome reporting (Reporting bias). Each domain had three possible judgments: "low risk", "high risk" or "unclear risk". A third author (DS) was consulted to resolve disagreements on risk of bias.

### Measures of treatment effect

The main comparison was between Ghrelin and control/placebo. The effect measures of treatment were relative risk (RR) for dichotomous variables and mean difference (MD) for continuous variables with 95% confidence intervals. When studies measured the same outcome with different measurement scales; standardised mean difference (SMD) was applied.

### Assessment of heterogeneity

We assessed heterogeneity amongst the studies by examining the forest plots for the overlap of confidence intervals. We analyzed the statistical heterogeneity through Chi^2^ test (P value < 0.10) and quantified through I^2^ and T^2^ statistics. Heterogeneity was regarded as substantial if in the χ² test for heterogeneity there was either I² more than 50%, T² more than zero, or P value < 0.10.

### Subgroup analysis and investigation of heterogeneity

During extraction of data, particular focus was put on the following aspects in order to check whether subgroup analyses would be appropriate for dose, duration and form of Ghrelin. We performed meta-analysis using a random-effects model. If heterogeneity was significant (P< 0.10), we explored the existence of qualitative differences through subgroup analyses and sensitivity analyses.

### Data synthesis

We performed meta- analysis using Review Manager 5.3 [41]. When we came across data from studies with similar comparison groups; we synthesized data using the Mantel-Haenszel method to obtain pooled, weighted risk ratios in random-effects.

Sensitivity analysis: We conducted sensitivity analyses to investigate the robustness of the results and to explore the effect of the study quality for each comparison by excluding trials rated as ‘High risk’ and restricting analysis to those trials rated as 'low risk’.

### Grading the quality of evidence

We used the GRADE approach cautiously to interpret the results [42]. Using the GRADE Profiler software; we imported the data from Review Manager 5.3 to create 'Summary of findings' tables and used it to guide our conclusions and recommendations. We assessed the quality of Evidence by grading the quality of the studies at four levels as “very low”, “low”, “moderate” and “high” on the basis of risk of bias, inconsistency, indirectness, imprecision and publication bias. Quality ratings were made separately for each outcome. On the basis of study limitations; review authors made an overall judgement on whether the quality of evidence for an outcome warrants downgrading. The GRADE tables were revised as per the need of animal studies. As the domain of indirectness is only applicable and can be assessed solely with the human subjects; by default; we downgraded the quality of evidence by 1 (as serious indirectness). Also; the quality rating was downgraded to one level if the evidence was classified as ‘serious’ and to two levels if it was classified as ‘very serious’. However, where potential limitations were not likely to lower confidence in the effect estimate, the evidence was not downgraded.

## Results

**Fig 1:**
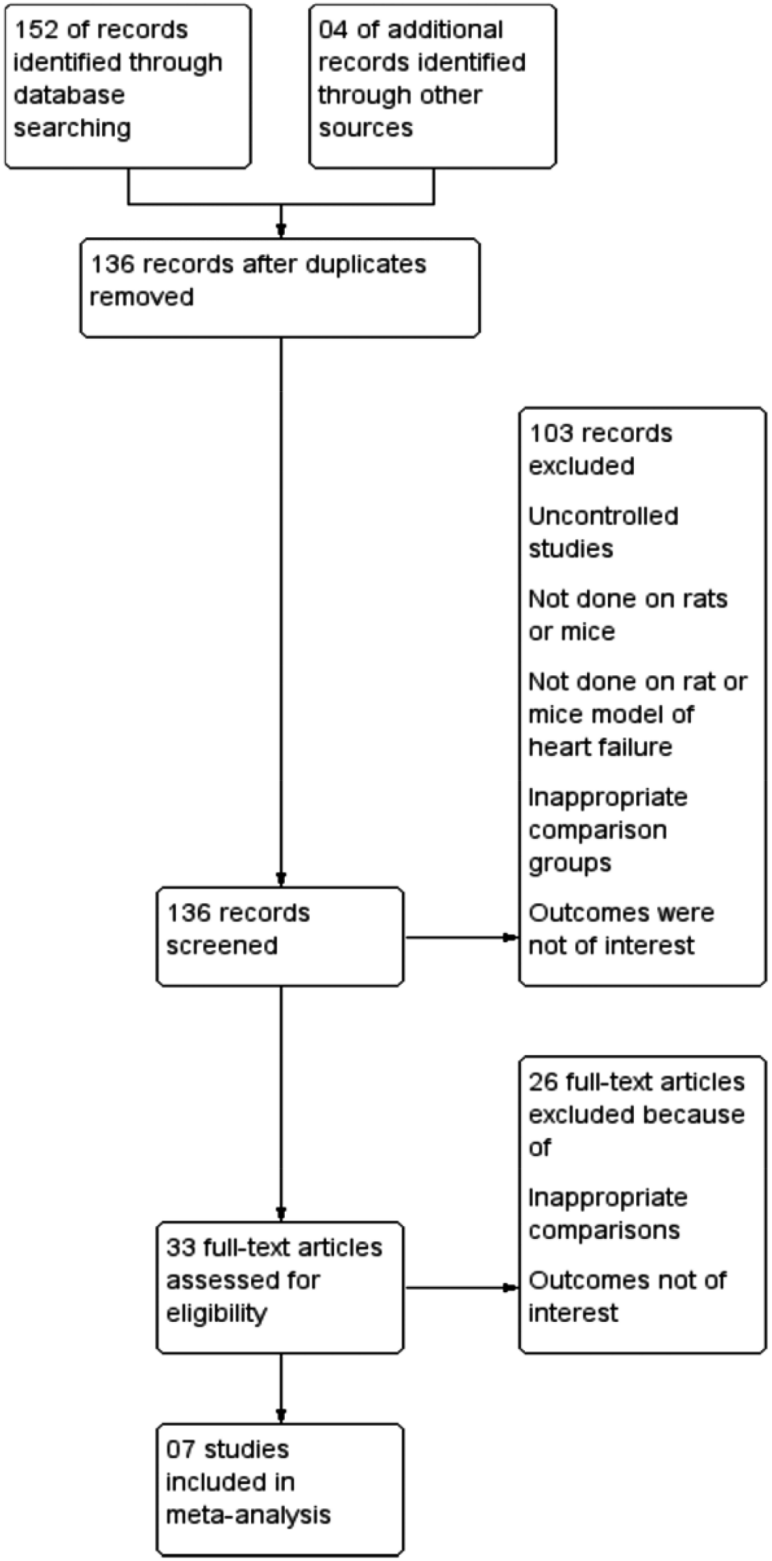
PRISMA Study flow diagram.

Description of studies: Our search strategy identified 136 references that described 33 pertinent studies, including two conference abstracts and two studies ahead of publication. Thirty-six studies were done on animals like rats, mice, pigs and rabbits. The studies were excluded on the basis of criterias mentioned in Fig 1[10, 19, 20, 24, 26, 27, 29, 30, 32, 35, 37, 43–49].

Seven studies met the inclusion criteria and were included in this review in Fig 1 [31, 50–55].

Three studies were carried out in China [51, 53, 54], two in Japan [31, 55] and one study each in Germany [50] and New Zealand [52]. Sample size ranged from 36 to 64 rats with a total of 325 rats included in seven studies. Five studies were done on Sprague—Dawley rats [50–54], one trial on C57BL/6J mice [31] and one study was done on Wistar rats [55]. The rats/mice weighed between 200–340 grams. [31, 50, 52, 55] and by injection of Isoprenaline in three studies [51, 53, 54]. The controls were subjected to sham operation, which involved a thoracotomy and cardiac exposure without ligation of the coronary artery. In most of the HF models, Ghrelin was administered subcutaneously (sc) [50–55] except in one study where hexarelin (a ghrelin analogue) was administered orally [31]. The duration of administration of Ghrelin ranged from two days to four weeks. The daily dose in the studies ranged between 1nmol/kg/day to100 nmol/kg/day. Ghrelin was not used in addition to conventional treatment for HF in any of the included studies. One trial demonstrated the difference in effect between Acyl Ghrelin and Desacyl Ghrelin [53]. Reported outcomes included mortality, cardiac effects and haemodynamic effects (Table 1). The fundings for the included studies were by academic institutions, local governments and international aid agencies.

**Table 1:** Characteristics of the studies.

Authors	Akashi 2009[50]	Zhang 2013 [51]	Daryl 2012 [52]	Li 2006 [53]	Lin Chang 2004 [54]	Mao 2014 [31]	Nagaya 2001 [55]
Country	Germany	China	New Zealand	China	China	Japan	Japan
Animal Care and experimental Protocols	Ethics committee of the Landesamt fur Gesundheit und Soziales (LaGeSo)	Animal Management rule of the Ministry of Health, People’s Republic of China and the Guide for the Care and Use of Laboratory Animals and were approved by the Animal Care Committee of health Sciences Centre, Peking University	Animal ethics Committee of the University of Otago, New Zealand.	Animal Management Rules of the Ministry of Health of the People’s Republic of China	The Council on Animal Care at the Peking University	Animal Ethics Committee of the National Cerebral and Cardiovascular Centre Research Institute, Japan In accordance with the Guidelines of the Physiological Society of Japan	Ethical committee of the National Cardiovascular Center
Total study duration (days)	54	4	14	10	56	14	61
Animal Model	Male Sprague Dawley rats	Male Adult Sprague Dawley rats	Male Sprague Dawley rats	Male Sprague Dawley rats	Male Sprague Dawley rats	Male mice (background strain C57BL/6J)	Male Wistar rats
Weight of Animal Model	228.4±1.0 g	250±10 gm	Wt ~280-340g	Wt 250-300g	Wt 200-250g	Not mentioned	200–240 g
Total Number of animal model	64	36	33	30	47	34	57
Setting	Laboratory	Laboratory	Laboratory	Laboratory	Laboratory	Laboratory	Laboratory
Age	Not mentioned	Adult	8 weeks old	Not mentioned	Not mentioned	14–16 week	Not mentioned
Method of Induction of heart failure	Ligation of Left anterior descending coronary artery	Induced by Subcutaneous injection of isoproterenol (ISO).	Ligation of Left anterior descending coronary artery	Induced by isoproterenol (ISO)	Induced by isoproterenol (ISO)	Ligation of Left anterior descending coronary artery.	Ligation of Left coronary artery
Housing condition	Maintained under controlled conditions with respect to temperature and humidity and were housed on a 12 h light/12 h dark cycle with free access to standard rat chow and water	Housed under standard conditions (room temperature 20±1°C; humidity 60±10% lights from 6 am to 6 pm and given standard rodent chow and water freely.	All rats were on a 12-h light,12-h dark cyle at 25±1 C and provided with food and water ad libitum	All animals were maintained on normal chow, had free access to water, and were kept in conditions of 12h light/12 h dark cycle	Not Mentioned	The mice were housed in a 12-h light/12-h dark cycle at 25°C and provided food and water ad libitum	The surviving rats were maintained on standard rat chow
Intervention groups	3	4	3	4	5	3	4
Mode of administration	subcutaneously	subcutaneously	subcutaneously	subcutaneously	subcutaneously	Oral gavage	subcutaneously
Timing Outcome measured	After 28 days of initiation of therapy.	Collected after 4 days of supplementation of ghrelin or placebo	Collected after 14 days	Collected after 10 days	Collected after 12 hours after the last injection	Collected on day 14 after induction of MI or sham operation	Collected after 3 weeks of treatment with ghrelin or placebo
Intervention	1.Sham (n = 15) 2.Placebo(n = 18) 3.GL (n = 17); 50nmol/kg/day, subcutaneously, TID for 28 days 4.GH (n = 19); 100nmol/kg/day, subcutaneously, TID for 28 days	1.Control (n = 9); saline; 2 mL/kg/d; od for 2 days. 2.ISO (n = 11); ISO 20mg/kg/d; od for 2 days. 3.ISO + ghrelin (n = 9); Ghrelin 10–8 mol/kg/d, subcutaneously; bid for 2 days. 4.ISO + metformin (n = 7); Metformin 250 mg/kg/d; x subcutaneously; bid for 2 days	1. Sham. 2.MI+ Saline(n = 7) Saline (0.3 ml = MI +Saline), 3.MI+ Ghrelin (n = 9) One bolus dose of 150 μg/kg ghrelin subcutaneously within 30 min of the infarct procedure	1.ISO Group (n = 7) 20,10 and 5 mg/kg, subcutaneously on day 1, 2 and 3 resp and 3 mg/kg for next 7 days. 2.Control group (n = 7) Normal saline, subcutaneously for 10 days. 3.ISO + Ghrelin (n = 7) 100μg/kg, BID, subcutaneously for 10 days. 4.ISO + DAG (n = 7) 100μg/kg, BID, subcutaneously for 10 days.	1.Control (n = 7) 0.9% NaCl; bid; subcutaneously for 2 days. 2.Ghrelin (n = 7) Ghrelin of 10 nmol/kg/d; bid; subcutaneously for 2 days. 3.ISO group (n = 11); ISO of 40 mg/kg/d; bid; subcutaneously for 2 days. 4.ISO+GL group (n = 11); ISO+ ghrelin of 1 nmol/kg/d; bid; subcutaneously for 2 days. 5.ISO+GH group (n = 11); ISO+ ghrelin of 10 nmol/kg/d; bid; subcutaneously for 2 day	1.Sham(n = 10); 2.Vehicle; Oral lavage. Vehicle (n = 24); Vehicle Oral gavage 30 minutes after infarct procedure. 3.Hexarelin (n = 24); Hexarelin 600μg/ mouse; Oral gavage 30 minutes after infarct procedure	1.Sham Placebo (n = 13); 100 μg/kg saline; BID; subcutaneously; 21 days. 2.Sham-Ghrelin (n = 13); 100 μg/kg ghrelin BID; subcutaneously; 21 days. 3.CHF-Placebo (n = 15); 100 μg/kg saline BID; subcutaneously; 21 days. CHF-4.Ghrelin (n = 16); 100 μg/kg ghrelin; BID; subcutaneously; 21 days
Outcomes Measures	Mortality, MABP, CO, EF, LV dP/dT max, LV dP/dT min, LVDD, LVFS	Mortality, HR,MABP, +LV dP/dT max, -LV dP/dT max, LVEDP, LVESP	Mortality, HR,CO, SV, EF, +LV dP/dT max, -LV dP/dT max, LVEDP, LVESP	+LV dP/dT max, -LV dP/dT max, LVEDP, LVESP	Mortality, HR,MABP, +LV dP/dT max, -LV dP/dT max, LVEDP	CO, SV, EF, +LV dt/dmax, -LV dt/dmax, LVEDP	Mortality, HR, MABP, LVEDP, LVESP
Funding	Non-Funded	National Natural Science Foundation of China	University of Otago, New Zealand. Ministry of Education, Culture, Sports, Science, and Technology of Japan. Alumni Association of Faculty of Medicine, Kagawa University. Banyu Life Science. Foundation International. ONO Medical Research Foundation. Takeda Science Foundation.	Non-Funded	Non-Funded	Ministry of Education, Culture, Sports, Science, and Technology of Japan. Takeda Science Foundation; Mochida Memorial Foundation for Medical. Pharmaceutical Research, Daiwa Securities Health Foundation. Senri Life Science Foundation	Ministry of Health, Labor, and Welfare; Uehara Memorial Foundation; Science Frontier program of MECSST,NEDO; Promotion of Fundamental Studies in Health Science

**Fig 2:**
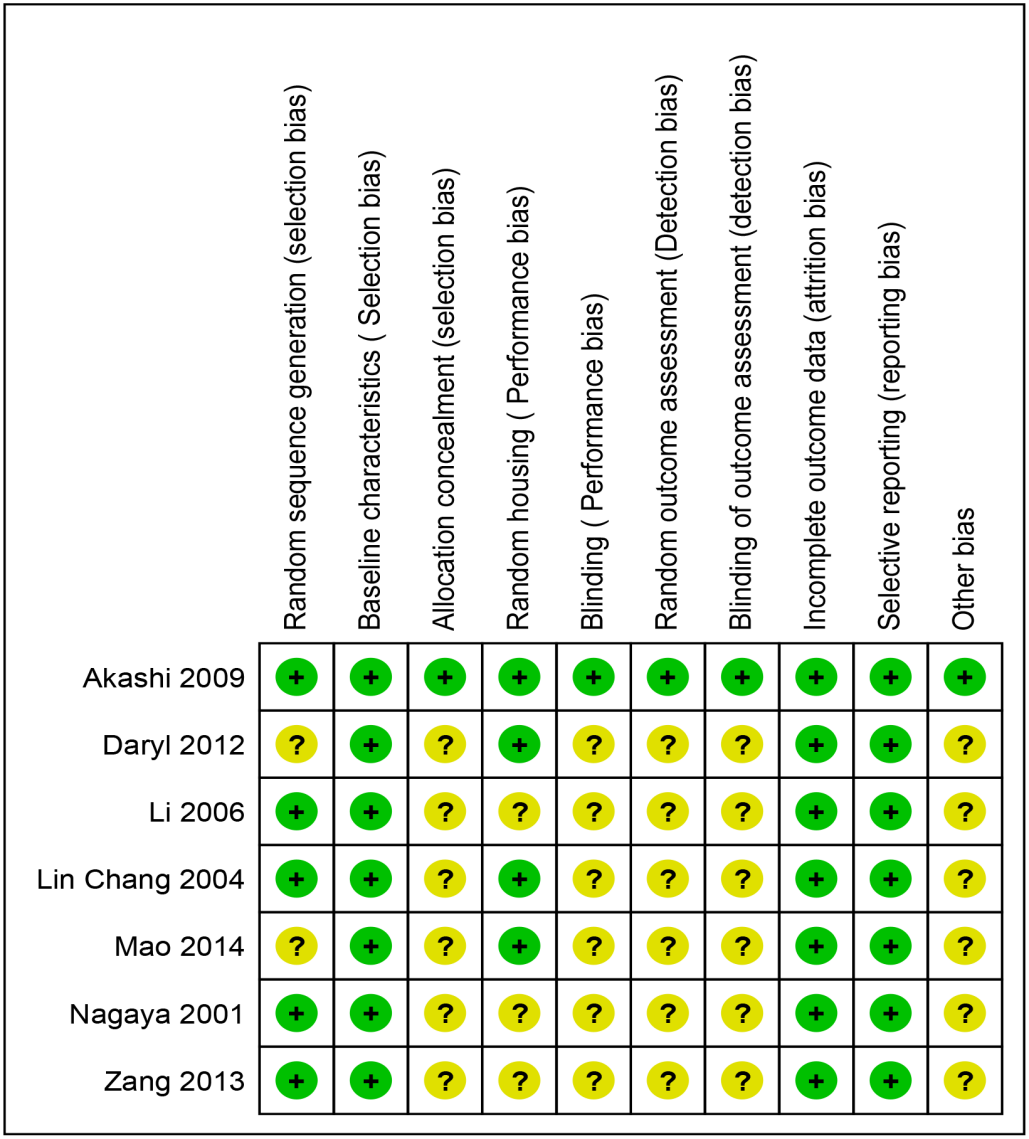
SYRCLE's Risk of bias summary: review authors' judgements about each risk of bias item for each included study [31,50,51,52,53,54,55].

**Fig 3:**
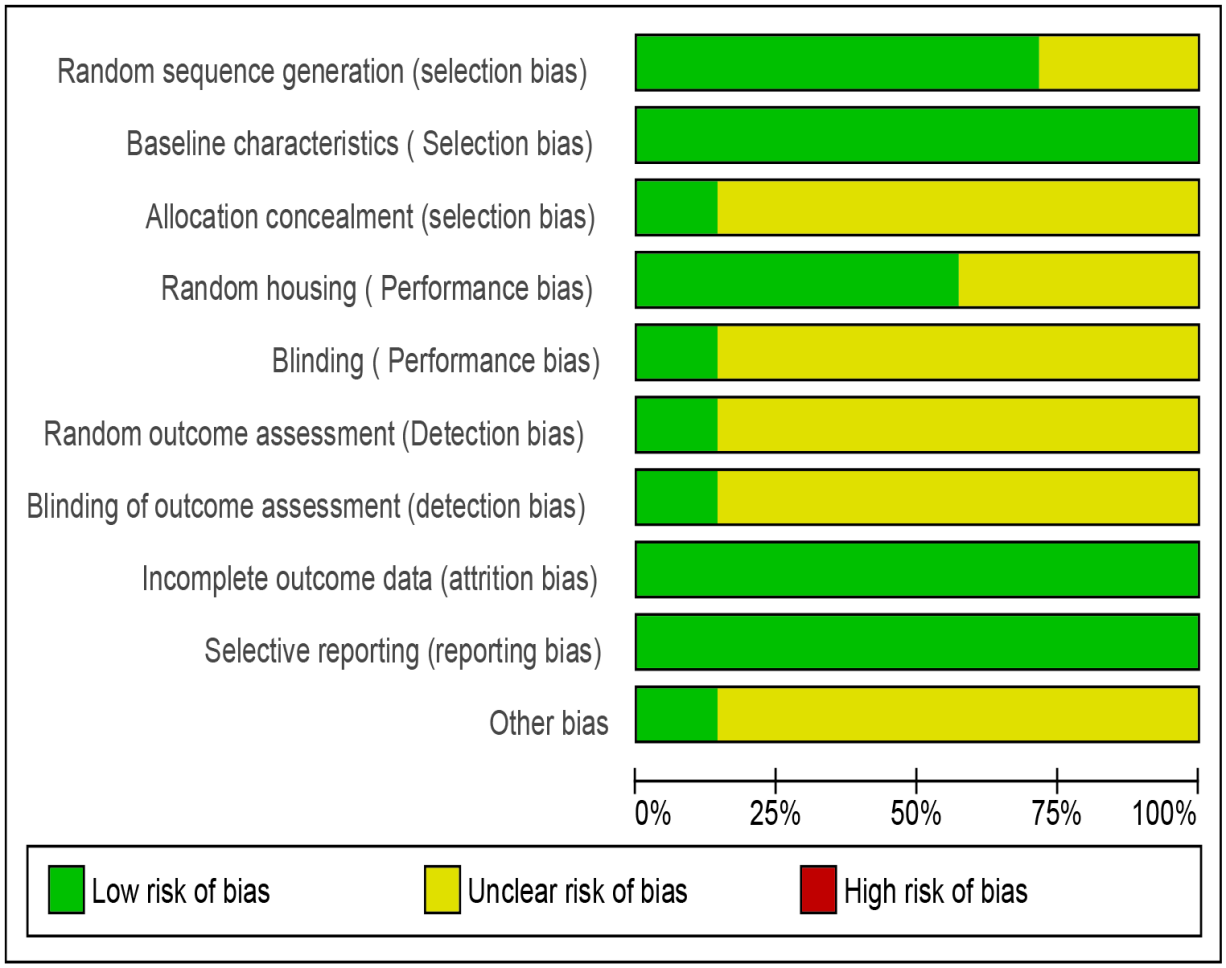
SYRCLE’s Risk of bias graph: review authors' judgements about each risk of bias item presented as percentages across all included studies [31,50,51,52,53,54,55].

Risk of bias in included studies: The risk of bias in the included studies was assessed with the SYRCLE tool for assessing risk of bias in animal studies [40]. Risks of bias in different domains for each included study is summarized in Fig 2 and each risk of bias item is presented as percentages across all included studies in Fig 3. The studies were at low risk bias for baseline characteristics, incomplete outcome data and selective reporting. Details of allocation concealment, random housing conditions, blinding of the care-giver/ investigator/ outcome assessor and random outcome assessment were poorly described across majority of the studies[40]. Assessment of baseline characteristics between the experimental and control groups did not reveal any significant difference (by p-value).

### Effects of interventions

#### Synthesis of results

While comparing Ghrelin with control/ placebo, a total of 325 rats and mice were analyzed across seven studies. We evaluated the effect of Ghrelin on mortality and different cardiovascular parameters.

#### Mortality

**Fig 4:**
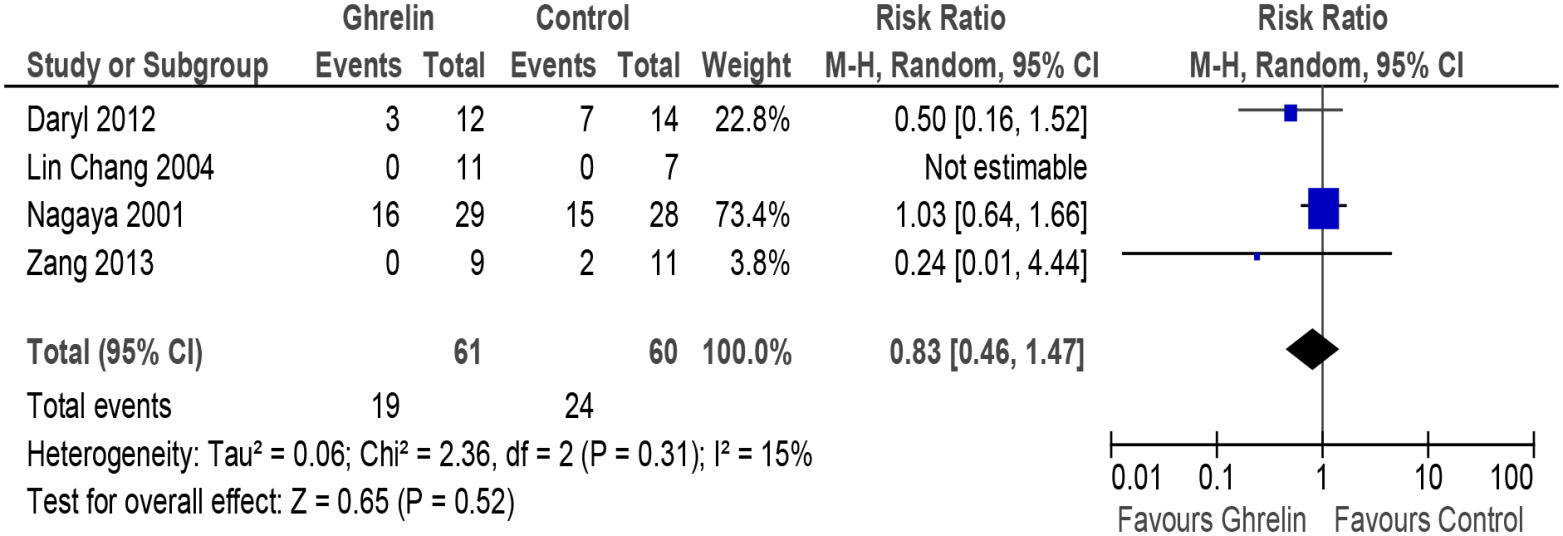
Forest plot of comparison: Ghrelin vs Control, outcome: Mortality [51,52,54,55].

The meta-analysis revealed that the mortality in Ghrelin group was 31.1% and in the control group was 40% (RR 0.83, CI 0.46 to 1.47, P = 0.52, n = 121) (Fig 4 and Table 2) i.e Ghrelin group had 68 fewer deaths per 1000 (from 216 fewer to 188 more) as compared to the controls.

**Table 2:** Summary of findings GRADE profile.

	Quality assessment							№ of patients		Effect			
	№ of studies	Study design	Risk of bias	Inconsistency	Indirectness	Imprecision	Other considerations	Ghrelin	Control	Relative (95% CI)	Absolute (95% CI)	Quality	Importance
Mortality	4 (51, 52, 54, 55)	randomised trials	serious ^1^	not serious	serious ^2^	very serious ^3^	none	19/61 (31.1%)	24/60 (40.0%)	**RR 0.83**(0.46 to 1.47)	68 fewer per 1000 (from 188 more to 216 fewer)	⨁◯◯◯VERY LOW	
									34.1%		58 fewer per 1000 (from 160 more to 184 fewer)		
Mean Arterial Blood Pressure	4 (51, 52, 54, 55)	randomised trials	serious ^1^	not serious	serious ^2^	not serious	none	59	54	-	MD **1.38 lower** (5.16 lower to 2.41 higher)	⨁⨁◯◯ LOW	
Heart rate	4 (50–52, 54)	randomised trials	serious ^1^	not serious	serious ^2^	not serious	none	56	58	-	MD **13.11 higher** (1.14 higher to 25.08 higher)	⨁⨁◯◯ OW	
Cardiac output	3 (31, 50, 52)	randomised trials	serious ^1^	not serious	serious ^2^	not serious	none	52	49	-	SMD **0.28 higher** (0.24 lower to 0.8 higher)	⨁⨁◯◯ LOW	
Left Ventricular End Diastolic Pressure (LVEDP)	5 (31, 51, 52, 54, 55)	randomised trials	serious^1^	not serious	serious ^2^	not serious	none	61	53	-	MD **2.45 lower** (4.46 lower to 0.43 lower)	⨁⨁◯◯ LOW	
Left ventricular End Systolic Pressure (LVESP)	4 (31, 47, 51, 55)	randomised trials	serious ^1^	not serious	serious ^2^	very serious ^5^	none	42	39	-	MD **1.48 higher** (3.86 lower to 6.82 higher)	⨁◯◯◯ VERY LOW	

MD—mean difference, SMD- standardized mean difference, RR—relative risk

^1.^ Most of the studies are not reported how they have generated the random code

^2.^ This domain is only applicable and can be accessed with human subjects

^3.^ The Confidence Interval is too wide

^4.^ The confidence Interval is too wide and the effect is in opposite direction.

#### Heart rate

**Fig 5:**
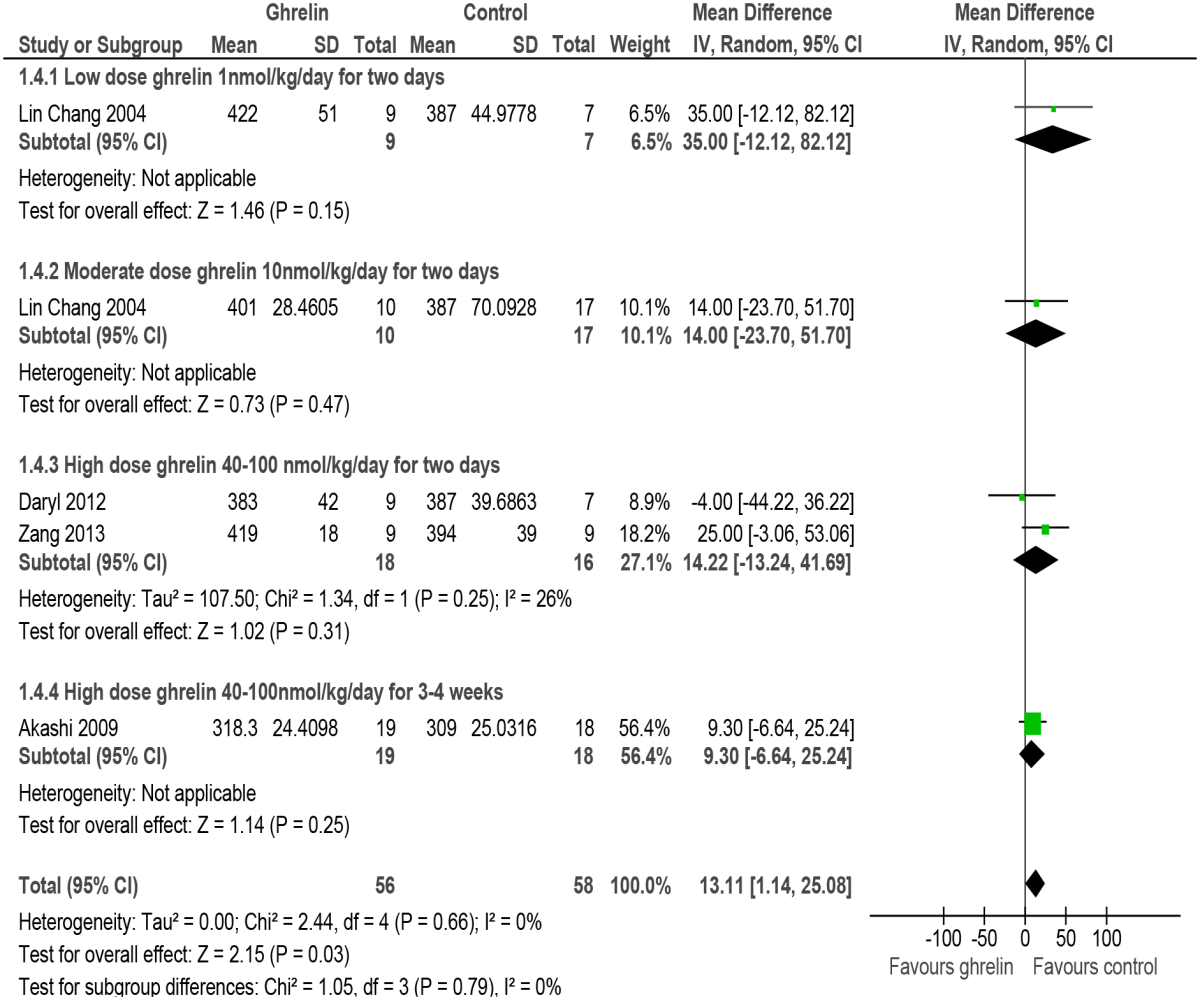
Forest plot of comparison: Ghrelin vs Control, outcome: Heart rate [50,51,52,54].

The meta-analysis of studies reporting data on mean heart rate [50–52, 54] indicated an increase in this outcome in rats/mice receiving Ghrelin as compared with rats/mice receiving placebo (MD 13.11, 95% CI 1.14 to 25.08, P = 0.66, n = 114). There was no evidence of heterogeneity amongst the studies (P = 0.66, I^2^ = 0%) (Fig 5, and Table 2).

#### Mean arterial blood pressure

**Fig 6:**
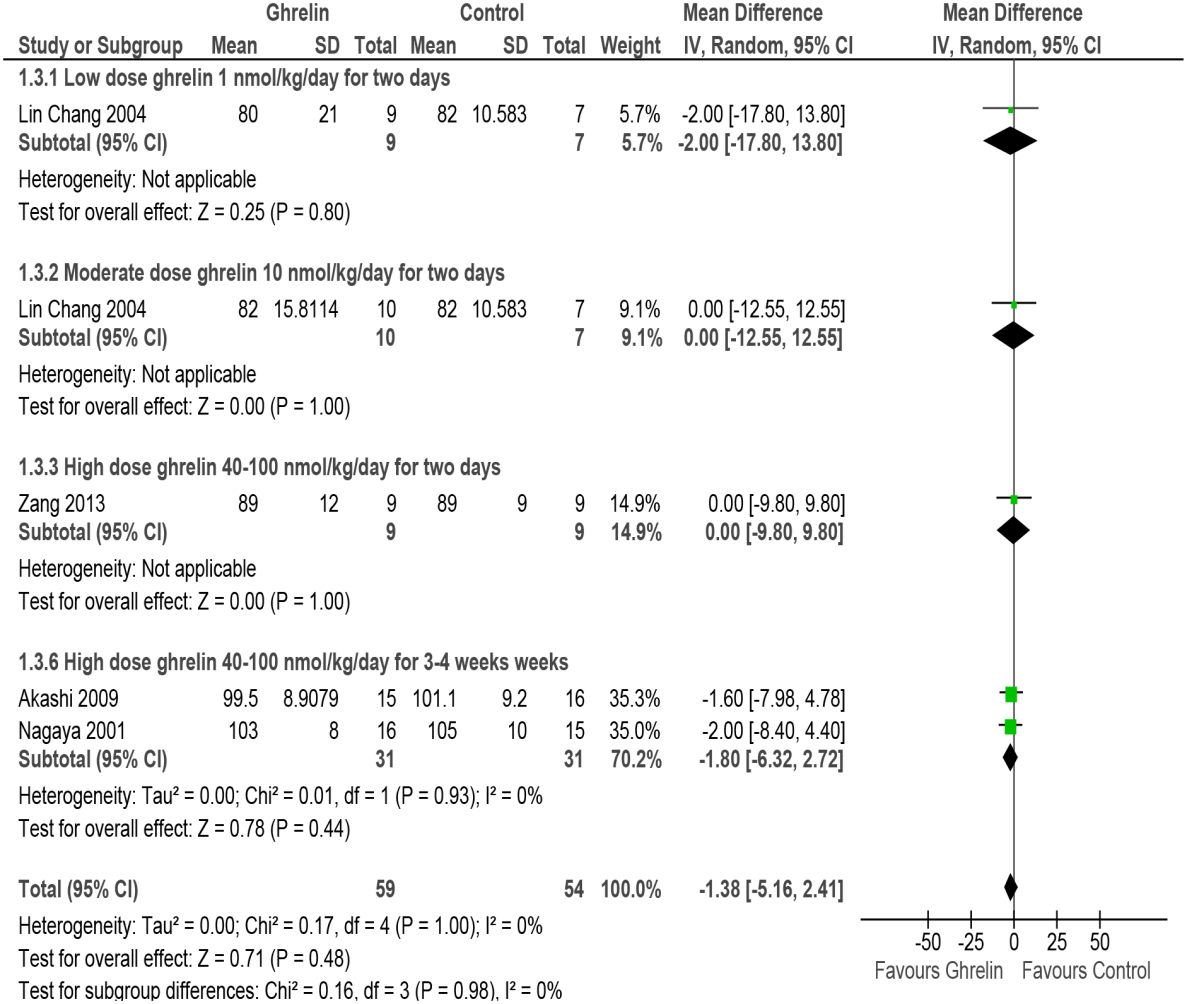
Forest plot of comparison: Ghrelin verses Control, outcome: Mean arterial blood pressure [50,51,54,55].

The meta-analysis of studies assessing data on mean arterial blood pressure [50, 51, 54, 55] showed an decrease in rats/mice put on Ghrelin as compared with those receiving placebo (MD -1.38, 95% CI -5.16 to 2.41, P = 0.48, n = 113). Subgroup analysis was carried out for low dose, moderate dose, high dose of Ghrelin for two days and high dose Ghrelin for 3–4 weeks though there was no evidence of heterogeneity amongst the studies (P = 0.98, I^2^ = 0%) (Fig 6 and Table 2).

#### Cardiac output

**Fig 7:**
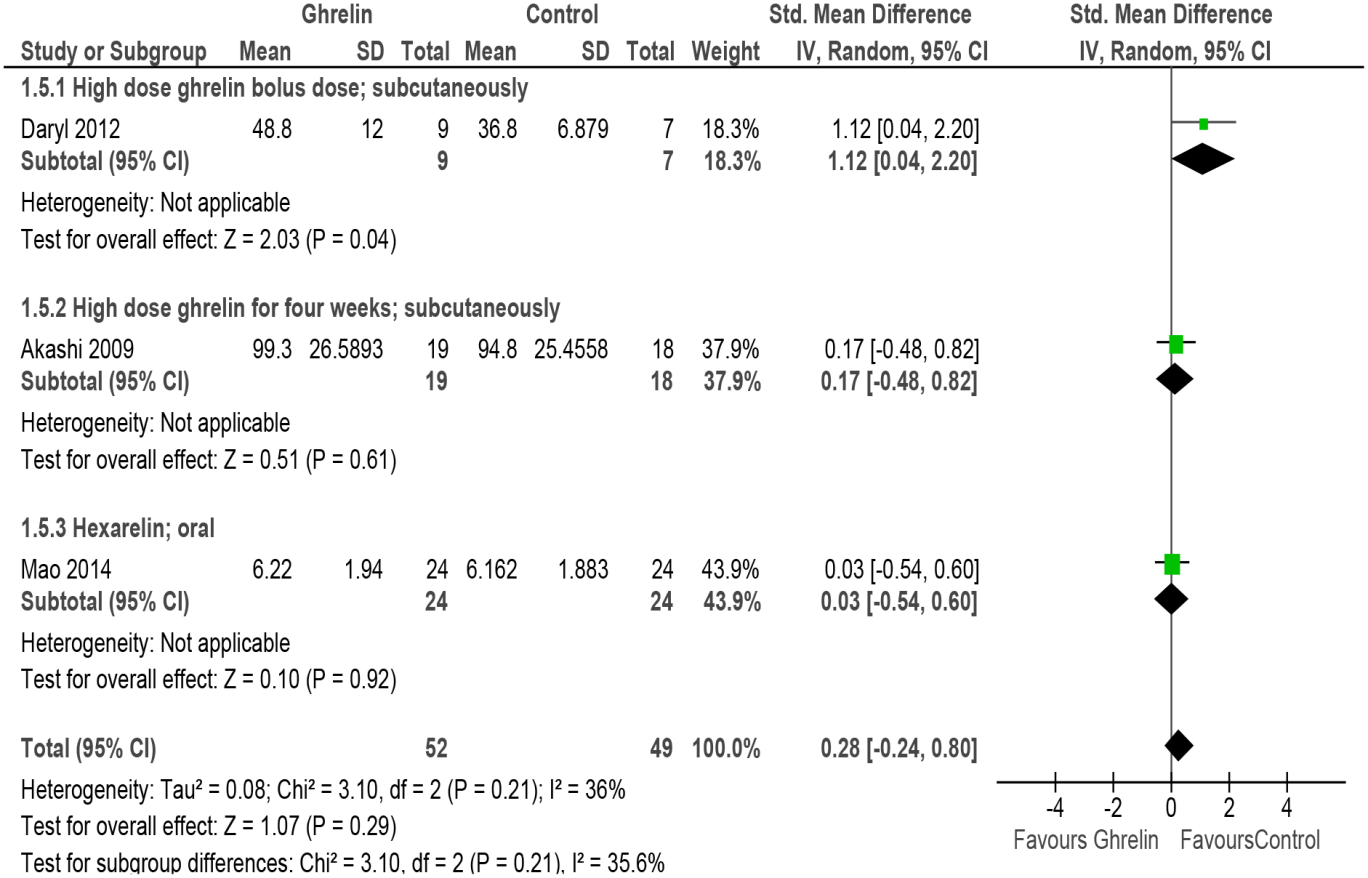
Forest plot of comparison: Ghrelin verses Control, outcome: Cardiac output [31,50,52].

Three studies evaluated the outcome of cardiac output [31, 50, 52] and the meta-analysis indicates that there is an insignificant change in cardiac output (SMD 0.28, 95% CI -0.24 to 0.80, P = 0.29, n = 101). There was evidence of mild heterogeneity amongst the studies (P = 0.21, I^2^ = 36%). Subgroup analysis was done for high dose Ghrelin (bolus dose, sc), high dose Ghrelin (4 weeks, sc) and Hexarelin (oral) (Fig 7 and Table 2).

#### Ejection fraction

**Fig 8:**
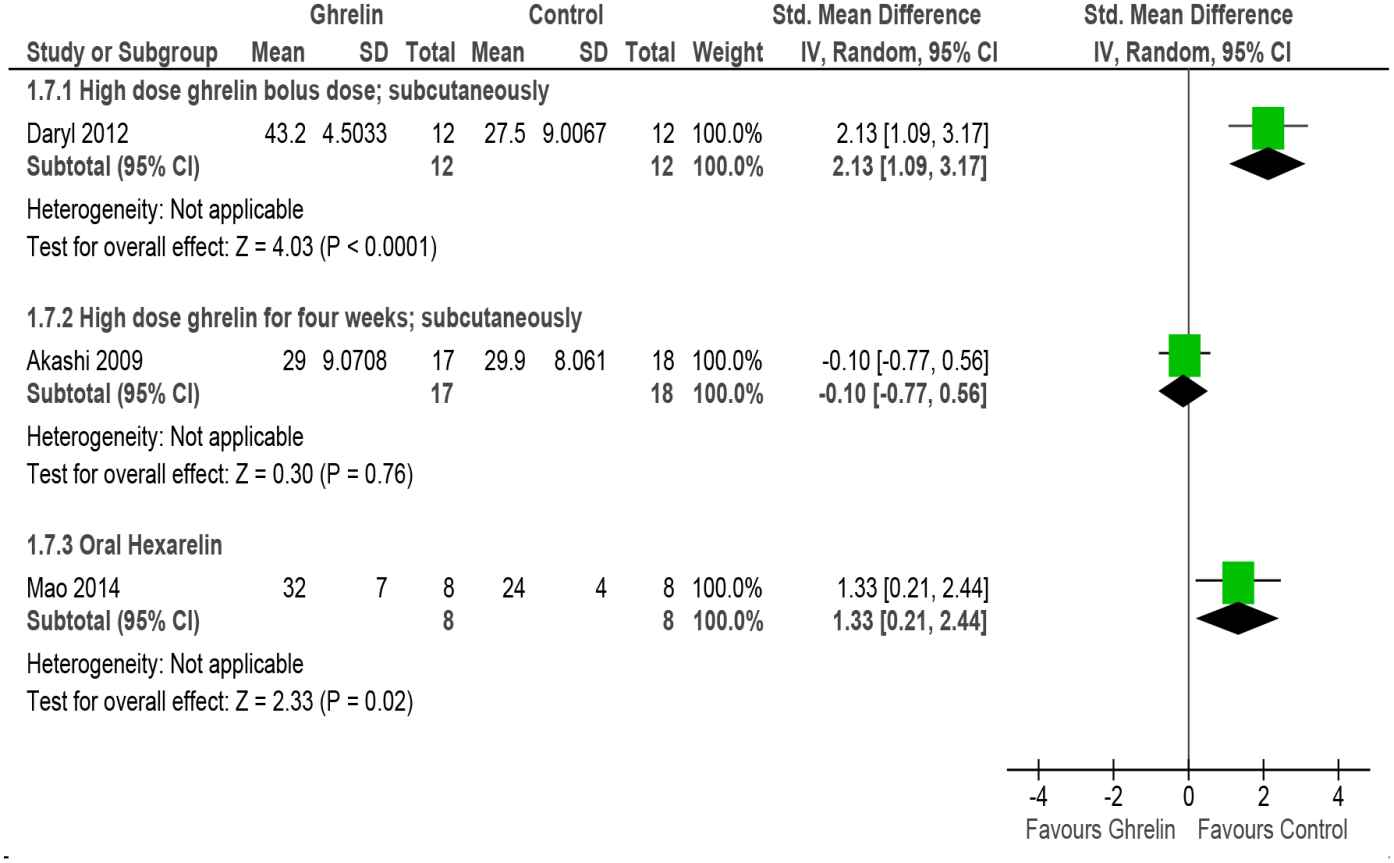
Forest plot of comparison: Ghrelin verses Control, outcome: Ejection fraction [31,50,52].

The meta-analysis of the three studies (31, 50, 52] with 75 rats/mice models of HF reporting data on ejection fraction revealed high degree of heterogeneity (P = 0.0008, I^2^ = 86%). Subgroup analysis was done for high dose (bolus, sc), high dose (4 weeks, sc) and oral Hexarelin. Tests for subgroup differences also revealed high heterogeneity (P = 0.0008, I^2^ = 85.9%) and so the data from the subgroups were not pooled (Fig 8 and Table 2). However; the overall effect was significant (P = 0.02)

#### LVESP

**Fig 9:**
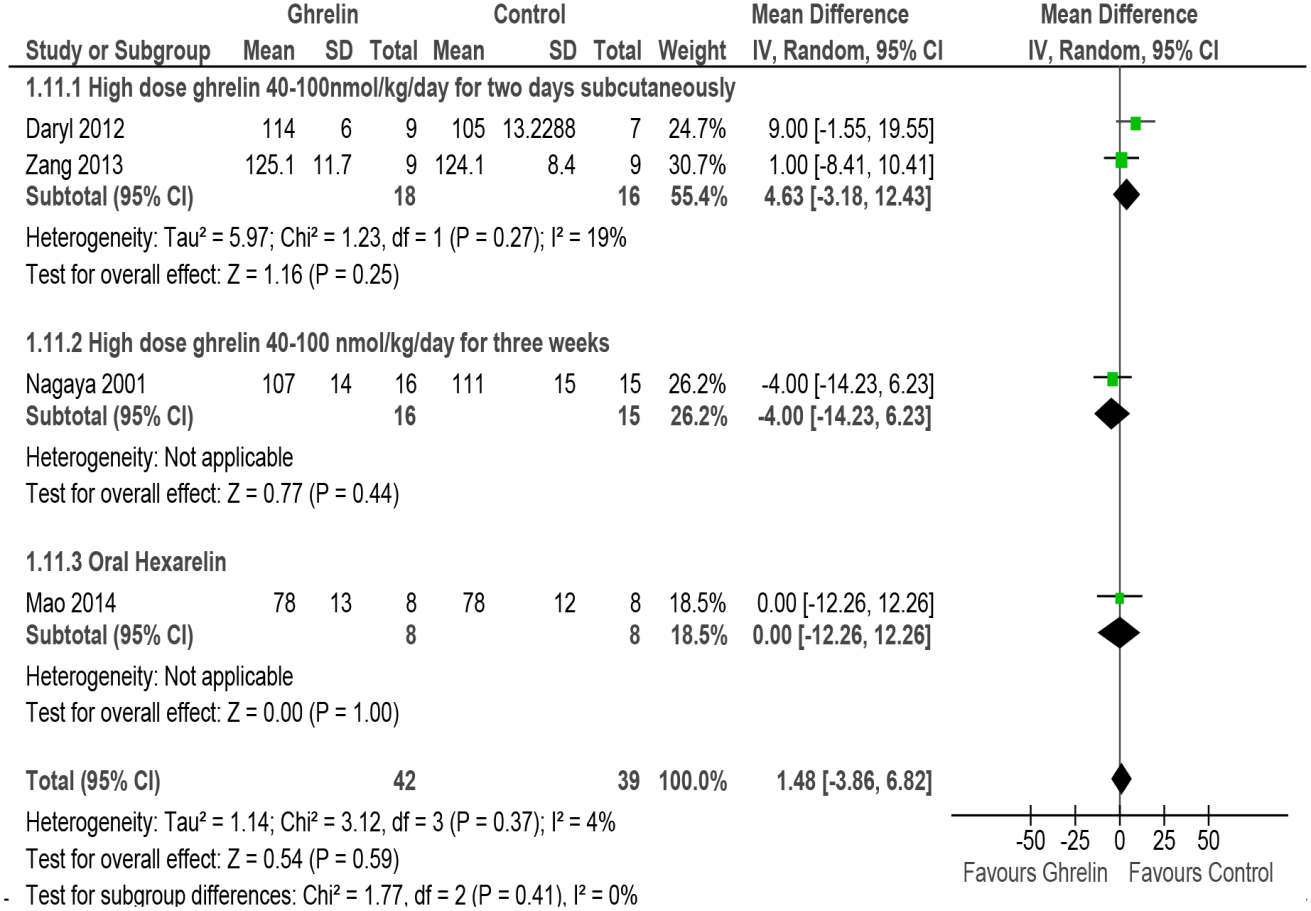
Forest plot of comparison: Ghrelin verses Control, outcome: LVESP [31,51,52,55].

Four studies reported the outcome of LVESP [31, 51, 52, 55]. The meta-analysis shows no effect of Ghrelin on LVESP (MD 1.48, 95% CI -3.86 to 6.82, P = 0.59, n = 81). There was no evidence of heterogeneity amongst the studies (P = 0.37, I^2^ = 4%). The studies differed in doses and so subgroup analysis was done for high dose Ghrelin (2 days, sc), high dose Ghrelin (3 weeks, sc) and oral hexarelin (Fig 9 and Table 2).

#### LVEDP

**Fig 10:**
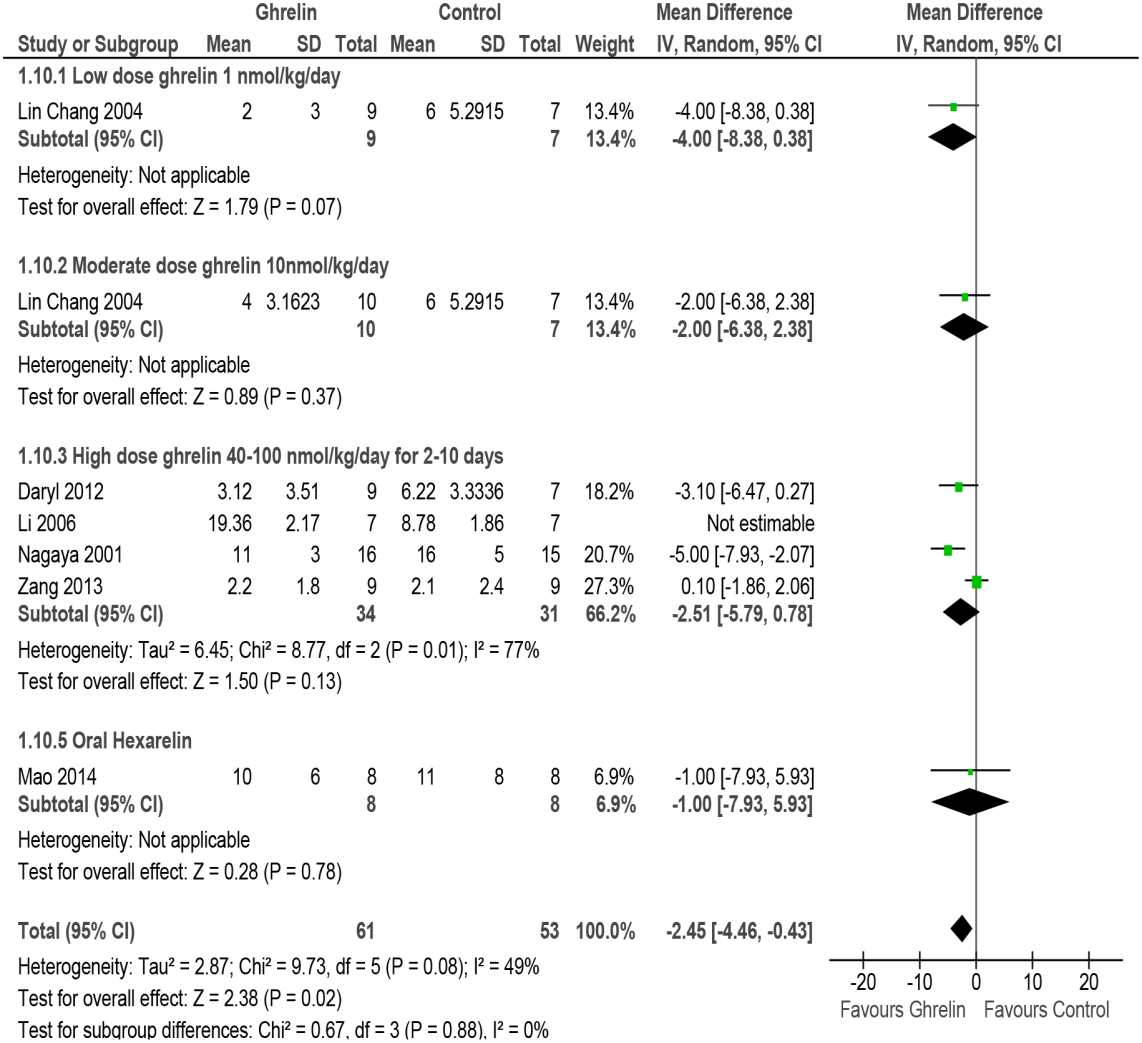
Forest plot of comparison: Ghrelin verses Control, outcome: LVEDP [31,51,52,53,54,55].

Meta-analysis of studies reporting effect of Ghrelin on LVEDP [31, 51–53, 55] showed a significant decrease (MD -2.45, 95% CI -4.46 to -0.43, P = 0.02, n = 114). Moderate heterogeneity was found in the analysis (P = 0.08, I^2^ = 49%) and so subgroup analysis was done for low dose Ghrelin, moderate dose Ghrelin, high dose Ghrelin and Hexarelin (oral) (Fig 10 and Table 2).

#### Quality assessment of evidence

The quality of the evidence as assessed with GRADEpro were graded between ‘Low’ to ‘Very Low’ as it was downgraded for risk of bias, indirectness and imprecision. The overall quality of the studies analyzed for mortality and LVESP was ‘Very Low’ while for parameters like heart rate, mean arterial blood pressure, cardiac output and LVEDP; it was graded as ‘Low’ (Table 2).

## Discussion

This review suggests that Ghrelin reduces mortality and improves cardiac function parameters in rats/mice models of HF. However, this conclusion is based on limited number of animal studies and needs to be interpreted with caution. As can be seen in the characteristics of included studies (Table 1); studies differed in strain/ species of rats/mice, method of induction of heart failure and doses of Ghrelin. Hence; subgroup analyses was carried out for majority of the outcome measures. Variables for subgroup analysis included dose, duration and type of Ghrelin and the results advocates that dose and not duration does seem to have an effect on the outcome measures. For comparison of Ghrelin versus control/placebo; beneficial effects of Ghrelin in the form of reduction of mortality, reduction of blood pressure, cardiac output and LVEDP were observed.

Although the mechanisms underlying the actions of Ghrelin on cardiovascular system remain uncertain; there are suggestions that its favourable effects are arbitrated through physiological actions like modulation of autonomic nervous system activity, improved energy balance, increased growth hormone levels and direct actions on cardiomyocytes [9, 36, 45].

Ghrelin is capable of modulating the sympathetic activity to the heart and the blood vessels [22]. In contrast to our analysis, a study has found that intra-cerebroventricular administration of Ghrelin led to significant lowering in HR which may be caused by reduction of sympathetic input to the heart [56]. Whether this inhibition of sympathetic input to the heart induced by Ghrelin is by direct effect on brainstem nuclei or by indirect effect via hypothalamus projections is still not clear and is a question of further research.

Ghrelin has been shown to enhance oxidation of free fatty acid and reduce oxidation of glucose in dogs with HF, thus partially correcting metabolic alterations in HF [45]. This novel mechanism might contribute to the cardioprotective effects of Ghrelin in HF. The studies show that in experimental animal models of HF, the administration of Ghrelin improves cardiac function and prevents the development of cardiac cachexia [57]. The administration of Ghrelin after early myocardial infarction (MI) reduced mortality and fatal arrhythmias [43, 44, 47]. In Ghrelin-deficient mice both exogenous and endogenous Ghrelin were protective against fatal arrhythmia and promoted remodelling after MI. In-vitro studies reveal that ghrelin increases the size, prolongs survival and protects the cardiomyocytes and endothelial cells against apoptosis and myocardial injury by stimulating Mitogen-activated protein kinases (p38-MAPK) activity and inhibiting AMP-activated protein kinase (AMPK) activity [58, 59]. In human aortic endothelial cells; ghrelin stimulates the production of nitrous oxide (NO) through activation of adenosine monophosphate-activated protein kinase (AMPK) and protein kinase B (Akt) [60]. Ghrelin system may play a vital role in regulating cardiac remodelling after myocardial infarction [49].

Heart failure; associated with an overexpression of mRNA for genes involved in ventricular remodelling, namely B-type natriuretic peptide (BNP) and atrial natriuretic peptide (ANP); causes increased release of BNP from ventricular cardiomyocytes [52, 61]. Thus; proBNP mRNA acts as an indicator of degree of stress in the ventricular walls. In rats; pre-treatment of Ghrelin before the induction of HF by isoprenaline (ISO) significantly reduces the expression of ventricular proBNP mRNA than with ISO alone. Histoanatomical data also indicates that ghrelin strikingly reduced the expression of BNP in mouse model of inherited dilated cardiomyopathy [62]. This indicates that ghrelin has a potential of ameliorating the increased wall stress and can protect heart against ischemia [51]. Also; in a human clinical trial on patients of HF, ghrelin correlated inversely with plasma Nt pro-BNP [63]. It has been demonstrated that administration of Ghrelin inhibits the inflammatory response in HF by bringing about a decrease in protein levels of tumor necrosis factor-alpha (TNF-alpha), interleukin (IL)-1beta and expression of matrix metalloproteinase (MMP)-2 and MMP-9 [64].

Lung weight/ tibial length higher in ghrelin-knockout (KO) mice and in models of HF [43]. Treatment with hexarelin or ghrelin in mice models of HF or ghrelin-knockout (KO) mice improved cardiac function as indicated by decreased lung weight:body weight and lung weight/tibial length compared with vehicle treatment [31, 43, 62].

Limited number of human clinical trials indicates a potential role for ghrelin in the management of HF [19, 22, 46]. The trials suggest that the plasma ghrelin levels are significantly lower in patients with HF than in controls and that their levels differed significantly with the severity of HF [55, 63]. Survival analysis indicates that elevated levels of ghrelin is a favorable prognosis for the patients with HF and can thus act as a new prognostic predictor for HF [63]. Administration of ghrelin in humans was shown to have beneficial hemodynamic effects [46]. Apart from improving muscle wasting, cardiac function, and exercise capacity; it also decreases systemic vascular resistance and exerts vasodilatory effects in patients suffering from HF [19, 57].

All this evidence suggests that Ghrelin can be a prospective target for modulation and treatment of cardiac remodelling [65] and a potential therapeutic target for cardiovascular disease. However more good quality preclinical (animal studies) should be undertaken to give more evidence.

## Summary of Evidence

The positive feature of the review is homogeneous study population (e.g., comparable/controlled housing conditions and animal characteristics). Only few studies provided data on our outcome variables. There were some methodological differences amongst the studies. Almost all the studies included in this review administered Ghrelin for relatively short periods without a long-term follow-up. Also, majority of the studies lacked description of randomization, sequence generation and details of drop-outs. Issues of biologic variation like biologic differences between rats and humans are likely to affect responsiveness to Ghrelin and this should be kept in mind while drawing conclusions. We have reported surrogate outcomes from animal studies, and are difficult to translate to the clinical setting. [66]. Also; we have graded the quality of evidence by GRADEpro software which has not yet been used for animal studies. Hence; this should be interpreted with caution. The animal studies are per-se subjected to variations. The results of these included studies are very different which can be due to differences in strain/ species of rats/mice, method of induction of HF or Ghrelin intervention. Therefore the overall strength of the evidence supporting the efficacy of Ghrelin is low though the discrepancies in the studies were dealt by doing subgroup analysis.

### Quality of the evidence

In general, animal studies in comparison to human RCT’s, have low internal validity as it is not yet a standard practice to randomize allocation of the animal to the intervention and the control arms and to blind the investigators and outcome assessors. However; some systematic reviews of animal studies show that a similar effect of an intervention can be found over a number of species/strains which suggests that there is a high probability that it can be extrapolated to humans.

## Conclusions

### Implications for practice

The existing data provides low quality evidence to suggest that administration of Ghrelin may lower mortality and may improve cardiac function parameters in rats/mice models of heart failure. However, there is paucity of evidence to support the incorporation of Ghrelin in the treatment of HF. Data from large, quality, randomized controlled trials are deficient. In view of the quality of trials; from which the data are extracted and the nature of the meta-analysis, the possible interaction should be interpreted with caution.

### Implications for future research

Future research should now be directed towards quality studies on all the different types of animal models for which publication checklists like gold standard publication checklist (GSPC) for animal studies needs to be used and followed [67]. Randomized trials on humans comparing various analogues in varying doses and duration of Ghrelin in HF patients can to be carried out. Data from good quality, large scale, multi-centric, long term, randomized controlled trials are needed to assess the efficacy of Ghrelin on HF. There is a need for a new tool to assess the quality of evidence for animal studies. Future trials should study the effect of Ghrelin on physiological as well as clinical outcomes.

## Supporting Information

S1 Table PRISMA Checklist. (DOCX)

S2 Table Search strategy. (DOCX)
