# Supplementary material for: Effect of Ghrelin on Mortality and Cardiovascular Outcomes in Experimental Rat and Mice Models of Heart Failure: A Systematic Review and Meta-Analysis
Source: PLoS One. 2015 May 27;10(5):e0126697. doi: 10.1371/journal.pone.0126697 (PMC4446297; doi:10.1371/journal.pone.0126697)
Supplement: S2 Table — (DOCX) [file pone.0126697.s002.docx]

| **Recent queries in Pubmed** | |
| --- | --- |
| Search | Query |
| #11 | Search ((((("Ghrelin"[Mesh]) OR ghrelin)) AND ((((((heart failure) OR cardiac failure) OR myocardial failure) OR cardiomyopathy)) OR heart failure[MeSH Terms]))) AND ((rat) OR mice) |
| #13 | Search ((((("Ghrelin"[Mesh]) OR ghrelin)) AND ((((((heart failure) OR cardiac failure) OR myocardial failure) OR cardiomyopathy)) OR heart failure[MeSH Terms]))) AND ((rat) OR mice) Filters: Other Animals |
| #10 | Search (rat) OR mice |
| #9 | Search ((("Ghrelin"[Mesh]) OR ghrelin)) AND ((((((heart failure) OR cardiac failure) OR myocardial failure) OR cardiomyopathy)) OR heart failure[MeSH Terms]) |
| #8 | Search (((((heart failure) OR cardiac failure) OR myocardial failure) OR cardiomyopathy)) OR heart failure[MeSH Terms] |
| #7 | Search ("Ghrelin"[Mesh]) OR ghrelin |
| #6 | Search (((heart failure) OR cardiac failure) OR myocardial failure) OR cardiomyopathy |
| #5 | Search heart failure[MeSH Terms] |
| #2 | Search "Ghrelin"[Mesh] |
| #4 | Search ghrelin |
